# Assessment of Sorbate and Benzoate Content in Mustard, Ketchup and Tomato Sauce by Sub-Minute Capillary Electrophoresis

**DOI:** 10.17113/ftb.59.03.21.7095

**Published:** 2021-09

**Authors:** Lais Morilla Pereira, Fabiana Della Betta, Siluana Katia Tischer Seraglio, Mayara Schulz, Priscila Nehring, Luciano Valdemiro Gonzaga, Roseane Fett, Ana Carolina Oliveira Costa

**Affiliations:** Department of Food Science and Technology, Federal University of Santa Catarina, Rodovia Admar Gonzaga 1346, 88034-001 Florianopolis, SC, Brazil

**Keywords:** food preservatives, sorbate and benzoate monitoring, food safety control, green analytical chemistry, sub-minute capillary electrophoresis

## Abstract

**Research background:**

Sorbate and benzoate are important preservatives in food products, but these compounds can also have genotoxic effects, causing health risks to consumers. In this regard, this study aims to determine the mass fractions of sorbate and benzoate in Brazilian samples of mustard, ketchup and tomato sauce using an adequately validated sub-minute capillary electrophoresis method.

**Experimental approach:**

In this study, sorbate and benzoate were evaluated in sauce samples by capillary electrophoresis using a simple sample preparation procedure. Previously, the method was validated according to Eurachem guidelines, and its greenness was assessed by Eco-Scale.

**Results and conclusions:**

The fitness for purpose of the method, as well as its suitability for the analysis of the studied matrices and its agreement with the principles of green chemistry were checked and confirmed. Also, according to our findings, among the 30 commercial samples assessed, six of them presented some mislabeling or non-compliance with European or Brazilian legislation, reinforcing the constant need for quality assessment and surveillance of food products.

**Novelty and scientific contribution:**

So far, there have been few studies related to investigating the preservatives such as sorbate and benzoate in mustard, ketchup and tomato sauce, highlighting the significance and contribution of the obtained results to the knowledge in the field.

## INTRODUCTION

Sorbic and benzoic acids and their salts play a significant role as preservatives in the food industry. Due to their capacity to inhibit yeasts, molds and bacteria, these additives are widely used in foods and beverages such as bread, sauces, soft drinks, cheese, juice and fermented products (*1*–*4*).

Due to the low solubility of sorbic and benzoic acids in water, potassium sorbate and sodium benzoate are preferably used since they have high water solubility (*4*, *5*). Each preservative is used in its optimum pH range, *i.e.* sorbates are effective at pH=6.5 or lower, and their antimicrobial activity increases as the pH decreases. At the same time, the benzoates are most effective in the pH range between 2.5 and 4.0 (*4*, *6*).

Until recently, the use of sorbate and benzoate was considered safe, although studies in cells and animals have evidenced that besides allergic reactions, their consumption may be related to the generation of carcinogenic and mutagenic compounds (*7*). In this sense, the maximum acceptable daily intake was established for benzoic acid and its salts at 5 and for sorbic acid and its salts at 25 mg per kg body mass per day (*8*).

In this regard, regulatory agencies established maximum levels for their application in food products, including sauces and the like, since these preservatives are widely used in these types of products, and these foods are commonly consumed daily. The European Commission established the mass fraction of 1000 mg/kg as the maximum limit for the sum of sorbate and benzoate in non-emulsified sauces, for example (*9*). The Brazilian legislation also authorizes these preservatives in some food products such as mustard and tomato sauce at the maximum mass fraction of 1000 mg/kg of sorbic and benzoic acids or their salts, which can be used either individually or in combination. For ketchup, only sorbic acid and its salts are allowed at the maximum mass fraction of 1000 mg/kg (*10*).

Considering the importance of monitoring sorbate and benzoate in food products to guarantee consumer health, several analytical methods to determine these analytes in foodstuff have been reported in the literature, and most of them are based on liquid chromatography (*2*, *4*, *11*, *12*). Despite the high sensitivity, the liquid chromatography methods present some drawbacks, especially in regard to the acquisition and operational costs, and in general, they are not in agreement with the concept of green analytical chemistry. Also, other methods are reported in the literature, including gas chromatography (*3*, *13*–*15*) and capillary electrophoresis (CE) (*16*–*19*). Therefore, it is important and essential to develop methods that are simple, quick and economical to determine sorbate and benzoate in food products. CE is recognized by its fast separation, and among the separation techniques is one that most fits to the principles of green analytical chemistry, because it mainly uses aqueous solvents, generally does not involve laborious and multi-step sample preparation and it requires reduced amounts of reagents and sample, resulting in an equally reduced amount of waste (*20*, *21*). Therefore, the use of green and validated CE methods is very promising for a fast and reliable evaluation of sorbate and benzoate in food products.

In this context, this study aims to: (*i*) determine sorbate and benzoate mass fractions in Brazilian mustard, ketchup and tomato sauce using a sub-minute capillary zone electrophoresis method, (*ii*) validate the method for these food matrices, and (*iii*) determine the greenness of the method using the Eco-Scale.

## MATERIALS AND METHODS

### Reagents and solutions

The reagents Tris(hydroxymethyl)aminomethane (TRIS), 2-hydroxyisobutyric acid (HIBA), and sorbic, benzoic and salicylic acids were purchased from Sigma-Aldrich, Merck (St. Louis, MO, USA). The stock solutions of TRIS and HIBA (components of the background electrolyte) were prepared in ultrapure water (Milli-Q, Millipore, Bedford, MA, USA) at the concentration of 100 and 125 mmol/L, respectively. The stock solutions of sorbic, benzoic and salicylic acids were prepared in methanol (Merck) at the concentration of 1000 mg/L and kept under refrigeration ((5±2) °C) until the analysis, when they were diluted to obtain the working concentrations.

### Instrumental equipment

The analyses were performed in a capillary electrophoresis (CE) system (model 7100; Agilent Technologies, Palo Alto, CA, USA) equipped with a diode array detector. The data acquisition and treatment software were supplied by the manufacturer (HP ChemStation®).

The electrophoretic separations were performed according to Costa *et al.* (*16*) in an uncoated fused-silica capillary of 32 cm (8.5 cm effective length×50 μm inner diameter×375 μm outer diameter). The short-end injection mode was applied in this study because it allowed a faster analysis with excellent separation of the compounds. The applied voltage was 30 kV, with positive polarity at the injection side, the temperature was maintained at 25 °C, and UV detection was conducted at 200 nm for benzoate and salicylate, and at 254 nm for sorbate. The background electrolyte was composed of 25 mmol/L TRIS and 12.5 mmol/L HIBA, at pH=8.1. Salicylate was used as the internal standard.

### Samples and sample preparation

A total of 30 samples of mustard (*N*=10), ketchup (*N*=10), and tomato sauce (*N*=10) from different brands were purchased in a local supermarket of Florianopolis, Santa Catarina state, Brazil.

The sample preparation procedure was modified from that proposed by Javanmardi *et al.* (*12*), who used an aqueous dilution followed by dispersive liquid-liquid microextraction (DLLM). In the original protocol, 5 g of sauce sample were diluted in 50 mL of water and centrifuged at 1784×*g* for 5 min. An aliquot of 5 mL of the supernatant was transferred to a test tube in which 750 mg NaCl and 1.2 mL acetone (disperser solvent) containing 250 µL chloroform (extraction solvent) were added. The mixture was homogenized for 2 min and centrifuged at 1784×*g* for 10 min. The sediment phase was quantitatively placed into another test tube and evaporated with nitrogen gas. The residue was redissolved in 100 µL mobile phase for high-performance liquid chromatography (HPLC) analysis. In the present study, a simple sample preparation based only on aqueous dilution was employed as follows: samples ((1.00±0.05) g) were weighed into polypropylene tubes, and 10 mL of ultrapure water were added. The tubes were sealed, and the resulting solution was stirred with a vortex (model 772; Fisatom, São Paulo, Brazil) for 1 min and centrifuged for 5 min at 9861×*g* (Mini Spin plus, Eppendorf, Hamburg, Germany). The supernatant was filtered through syringe filters (0.45 µm pore size, Millipore) and diluted in the proportion of 5:1 (*V*/*V*) with salicylate (internal standard) at the concentration of 120 mg/L before being injected at the CE system. Subsequent dilutions were performed when necessary. For all experiments (quantification and validation) the corrected peak area (*A*_analyte_/*A*_salicylate_) and the corrected migration time (*t*_m(analyte)_/*t*_m(salicylate)_) were considered.

All samples were prepared in three independent replicates and analyzed before the expiration date.

### Analytical validation and greenness assessment

The analytical method was originally proposed and validated by Costa *et al.* (*16*) to assess sorbate and benzoate in soft drinks, juices and teas. Regarding the application of this method to different matrices (mustard and tomato-based samples) and a change performed in the linear range, it was necessary, in this study, to reassess the validation parameters such as linearity, matrix effect, selectivity, precision and accuracy. The validation protocol followed the Eurachem guidelines (*22*). In addition, the greenness of the analytical procedures employed in this study was quantitatively determined using the Eco-Scale proposed by Gałuszka *et al.* (*23*).

## RESULTS AND DISCUSSION

### Capillary zone electrophoresis method and sample preparation adaptations

**Fig. 1:**
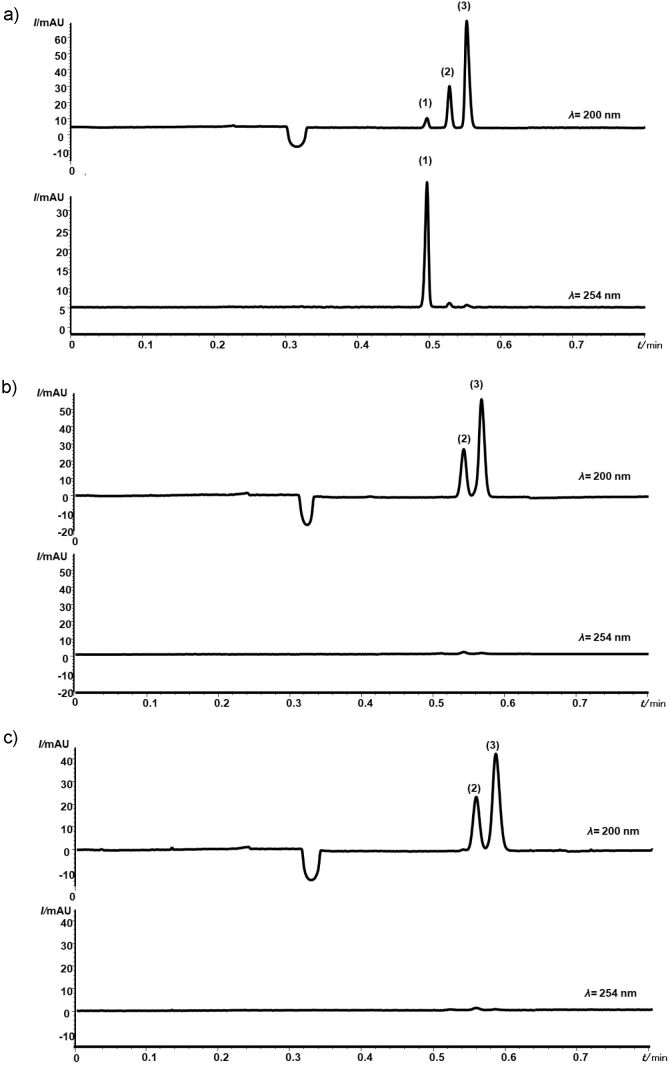
Electropherograms of: a) standard solution, b) mustard sample G, and c) ketchup sample I. Separation conditions: background electrolyte composed of 25 mmol/L Tris(hydroxymethyl)aminomethane and 12.5 mmol/L 2-hydroxyisobutyric acid, at pH=8.1; hydrodynamic injection of 5000 Pa (50 mbar) for 3 s, voltage of +30 kV, and capillary of 32 cm (8.5 cm effective length×50 μm inner diameter×375 μm outer diameter). Peaks: 1=sorbate, 2=benzoate, and 3=salicylate as internal standard

In this study, the capillary zone electrophoresis (CZE) method proposed by Costa *et al.* (*16*) for non-alcoholic beverages was successfully applied to determine sorbate and benzoate in mustard, ketchup and tomato sauce, as shown in Fig. 1.

This method allowed the separation of both ions and the salicylate in just 28 s. The total analysis time of the method, which includes flushing, vial permutation, injection and separation, was experimentally determined and took about 1.3 min, achieving an analytical throughput of approx. 40 samples per hour. Moreover, the high throughput of the CE method is noteworthy compared to chromatographic methods such as NMKL (Nordic Committee on Food Analysis) method 124 (*24*), in which the target analyte separation takes over 15 min.

As shown in Table 1 (*16*-*19*, *25*, *26*), different separation conditions and background electrolyte (BGE) composition can be used for sorbate and benzoate determination by capillary electrophoresis. However, the method proposed by Costa *et al.* (*16*) and employed in the present study allowed the separation of both analytes in less than 30 s. In contrast, separation times between 5 and 7 min were usually necessary when other separation conditions were applied. In addition, the limits of detection and quantification found by Costa *et al.* (*16*) were similar to those reported by the other studies indicating that the short separation time did not impact the sensibility of the method.

**Table 1:** Separation conditions of benzoate and sorbate by capillary electrophoresis methods

BGE and separation parameter	Separation time, LOD and LOQ	Method
BGE: 25 mmol/L Tris(hydroxymethyl)aminomethane and 12.5 mmol/L 2-hydroxyisobutyric acid, at pH=8.1 Short-end injection: 32 cm total length×8.5 cm effective length×50 μm inner diameter Voltage: +30 kV Temperature: 25 °C Detection: UV direct at 200 nm for benzoate and 254 nm for sorbate	30 s LOD: 0.3 mg/L sorbate, 0.9 mg/L benzoate LOQ: 1.1 mg/L sorbate, 1.3 mg/L benzoate	Our study, Costa *et al.* (*16*)
BGE: 20 mmol/L disodium hydrogen phosphate and 0.2 mmol/L sodium phosphate monobasic, at pH=8.5 Injection: 48.5 cm total length×40 cm effective length×75 μm inner diameter Voltage: +15 kV Temperature: 29 °C Detection: UV direct at 200 nm for benzoate and sorbate	6 min LOD: 0.7 mg/kg sorbate, 1.0 mg/kg benzoate LOQ: 2.2 mg/kg sorbate, 3.1 mg/kg benzoate	Petruci *et al.* (*17*)
BGE: 10 mmol/L histidine,100 mmol/L propanesulfonic acid, 0.2 mmol/L cetyltrimethylammonium bromide, and 10% methanol, at pH=5.8 Injection: 80 cm total length×71.5 cm effective length×75 μm inner diameter Voltage: -27 kV Temperature: not informed Detection: conductivity detection at 633 kHz	7 min LOD: 1.2 mg/L sorbate LOQ: 4.1 mg/L sorbate	Coelho *et al.* (*18*)
BGE: 10 mmol/L benzoic acid, 15 mmol/L histidine and 0.2 mmol/L cetyltrimethylammonium bromide, at pH=5.8 Injection: 75 cm total length×67 cm effective length×75 μm inner diameter Voltage: -25 kV Temperature: not informed Detection: UV direct at 250 nm for sorbate	5 min LOD: 0.009 mg/L sorbate LOQ: 0.03 mg/L sorbate	de Jesus *et al.* (*19*)
BGE: 20 mmol/L sodium tetraborate, at pH=9.3 Injection: 48.5 cm total length×40 cm effective length×75 μm inner diameter Voltage: -20 kV Temperature: 25 °C Detection: UV direct at 255 nm for sorbate	7 min LOD: 0.4 mg/L sorbate LOQ: 1.0 mg/L sorbate	Mesquita *et al.* (*25*)
BGE: 50 mmol/L borax, at pH=10.5 Injection: 30 cm total length x 20 cm effective length×50 μm inner diameter Voltage: -15 kV Temperature: 25 °C Detection: UV direct at 200 nm for sorbate and benzoate	3 min LOD: 0.4 mg/L sorbate and benzoate LOQ: 1.2 mg/L benzoate, 1.3 mg/L sorbate	Nowak (*26*)

BGE=background electrolyte, LOD=limit of detection, LOQ=limit of quantification

The successful application of the CZE method in this study also depended on the use of suitable sample preparation. The extraction of the analytes was based on modifications of the procedure proposed by Javanmardi *et al.* (*12*), who determined sorbate and benzoate in ketchup and other foods using the pre-preparation step followed by DLLM coupled with HPLC. Besides the non-utilization of DLLM, the modifications performed in the present study made the extraction procedure simpler and more appropriate for green chemistry by reducing the sample mass and the residue generation. In addition, the proposed method proved to be adequate to extract the analytes from the analyzed samples, eliminating the need to use one more step of cleaning and concentration of the analytes, such as in DLLM. Despite the use of minimum sample preparation steps, the peaks in electropherograms are well separated, as shown in Fig. 1b and Fig. 1c.

### Analytical validation

To ensure the fitness for purpose and the veracity of the data, it was necessary to reassess the validation parameters of the CZE method for the new food matrices investigated in this study.

#### Linearity and matrix effect

To assess the linearity, calibration curves of the standard solution of analytes and calibration curves of the matrix with internal standard addition were prepared. Each calibration curve was made in six equally spaced concentrations (5-30 mg/L) and three independent replicates, and randomly injected in the CE system.

Two matrix calibration curves were prepared, one for mustard and another one for ketchup, both prepared by standard addition to blank samples. Due to the similarities in the composition of ketchup and tomato sauce, we assume that the interfering is comparable and, therefore, just one curve was built for ketchup to represent all styles of tomato-based sauces. In order to reproduce accurately the presence of possible interfering of the matrix, the blank samples used were obtained under the extraction and dilution conditions described in the sample preparation paragraph of Materials and Methods section.

After the CE analysis and data acquisition, calibration curves were built by plotting the corrected peak area (sorbate/salicylate and benzoate/salicylate) *versus* concentration, and all curves had coefficient of regression R^2^>0.99. Residual plots were assessed by visual inspection, and no lack of linearity or heteroscedasticity was verified. The assumptions of normality (*27*), homoscedasticity (*28*), and independence (*29*) were confirmed, and no lack of fit was observed, confirming the linearity at this range of concentrations for standard solution and matrix calibration curves. The validation parameters are shown in Table 2.

**Table 2:** Analytical performance of the sub-minute capillary zone eletrophoresis method for the assessed matrices

Parameter	*N*	Sorbate	Benzoate
Linear range/(mg/L)	-	5 – 30	
Linearity – standard solution			
Slope	3	0.0210	0.0173
Intercept	3	0.0381	0.0334
R^2^	3	0.999	0.998
Linearity – mustard			
Slope	3	0.0226	0.0165
Intercept	3	0.0492	0.0832
R^2^	3	0.998	0.995
Linearity – ketchup			
Slope	3	0.0175	0.0153
Intercept	3	0.1007	0.1164
R^2^	3	0.996	0.997
Precision (RSD/%)			
Intra-day precision – *A*_corrected_	3	1.43	1.51
Intra-day precision – *t*(migration)_corrected_	3	0.18	0.04
Inter-day precision – *A*_corrected_	9	2.06	2.56
Inter-day precision – *t*(migration)_corrected_	9	0.50	0.21
Recovery/%			
*γ*(mustard)/(mg/L) 10	3	99.0±1.0	99.9±2.1
20	3	101.1±1.8	101.6±1.4
30	3	99.3±0.5	98.8±1.9
*γ*(ketchup)/(mg/L) 10	3	98.8±1.7	104.2±1.2
20	3	97.8±2.7	103.4±1.9
30	3	91.6±1.8	94.2±0.9

RSD=relative standard deviation, *A*=peak area

The matrix effect was assessed by comparing the slopes obtained for the standard solution and matrix calibration curves (mustard and ketchup). Differences were considered statistically significant at the 5% level (p<0.05) through the *t*-test bi-caudal. The presence of matrix effect was observed for the ions of sorbate (p=0.0002) and benzoate (p=0.01) in ketchup and sorbate in mustard (p=0.006). In this regard, all quantifications were performed using matrix calibration curves.

#### Selectivity, precision and accuracy

The selectivity was evaluated experimentally by injecting the analytes (sorbate, benzoate and salicylate) and the possible interferent (ascorbic acid) under the separation conditions. After the injection, the electropherogram was visually inspected, and the capacity of the method to accurately separate the analytes in the presence of the interferent was confirmed.

The parameter repeatability (intra-day) was assessed using three consecutive injections of a standard solution of the analytes (sorbate, benzoate and salicylate). At the same time, the intermediate precision (inter-day) was determined by injecting this standard solution three times for three consecutive days. The method precision was confirmed since the results for both intra- and inter-day precision were lower than 2.60% relative standard deviation (Table 2).

The accuracy was established by recovering blank samples fortified at three concentrations (10, 20 and 30 mg/L). The results for sorbate and benzoate in mustard ranged from 99.0 to 101.1% and 98.8 to 101.6%, respectively, while in ketchup, they ranged from 91.6 to 98.8% for sorbate and 94.2 to 104.2% for benzoate (Table 2). Therefore, these results indicated adequate method accuracy.

#### Analytical Eco-Scale for greenness assessment

The scientific community has shown a growing interest in the development of environmentally sustainable analytical methods. Several authors name their methods as ’environmentally friendly’, however, this affirmation often comes from an empirical denomination, since there is no clear evidence to support it. Recently, some approaches have been proposed to quantitatively determine this claim in analytical procedures. Some examples are the National Environmental Methods Index (NEMI) labeling, Analytical Greenness Calculator (AGREE), white analytical chemistry (WAC) and Eco-Scale (*30*).

The Eco-Scale is based on the assignment of penalty points to parameters of an analytical process that are not in agreement with the ideal green analytical method. These penalty points are subtracted from a base of 100, the value considered for the ideal green method. The penalty points should consider all aspects of the analytical procedure, from the type and amount of reagents applied, energy consumption, waste generation, and the level of occupational hazard to which the analyst is exposed (*23*, *31*).

As shown in Table 3, all the reagents and instruments used in the determination of sorbate and benzoate were considered to calculate the Eco-Scale. The proposed method was assigned 26 penalty points, resulting in an Eco-Scale score of 74, which is characterized as acceptable in terms of greenness. The use of reagents such as methanol and benzoic and sorbic acids resulted in important penalty points, which directly contributed to the obtained score for the method. However, parameters related to the occupational hazard and energy consumption were not significant, and the sample preparation steps also contributed to the greenness of the method since the procedure was based just on the sample dissolution, centrifugation and filtration.

**Table 3:** Green evaluation of the sub-minute capillary zone electrophoresis method according to the Eco-Scale

Aspect of the analytical procedure	Penalty point
Reagents	20
Energy consumption	0
Occupational hazard	1
Waste	5
Total	26
Eco-Scale	74

Recently, Nowak *et al.* (*30*) presented a new perspective for sustainable development in analytical chemistry named WAC as an analogy to the 12 principles of green analytical chemistry. The concept of WAC is based on a tool known as Red-Green-Blue (RGB) model, where red colour represents analytical efficiency, green is the environmental friendliness and safety, and blue stands for the economic and practical aspects. Figuratively, when red, green and blue are highly saturated, the white colour is obtained, which represents the ideal analytical method. The main idea of WAC assumes that an analytical method needs to balance between greenness and applicability.

The 12 principles of WAC are grouped into 4 red, 4 green and 4 blue principles: (*i*) red principles are scope of application, sensitivity (LOD and LOQ), precision, and accuracy; (*ii*) green principles are toxicity of reagents, amount of reagents and waste, energy consumption, and impact on human and animal health; and (*iii*) blue principles are cost-benefit, time-efficiency, requirement of personnel and infrastructure, and simplicity of operation.

Alternatively assessing our study in terms of the principles of WAC, we can point out that the CE method follows most of the principles of WAC, since it can be applied to simultaneously determine three analytes, it can be applied to several matrices (soft drinks, juices, teas, sauces), it has limits of detection and quantification consistent with the level of the analytes in the samples, and it has good precision and accuracy. Most of the reagents used are non-hazardous, and the hazardous one (methanol) was used at a minimum amount; the sample preparation and the CE method were developed in order to obtain a minimum consumption of reagents and waste generation. The energy consumption of the CE method is low, and by using correct disposal protocols the impact on the environment, humans and animals can be avoided. The cost-benefit of the method is great, the time of analysis is considerably short, allowing the analysis of several samples per hour. Besides, laboratory infrastructure requirement is low. The only aspect of the WAC that the CE method may not satisfy properly is the last blue principle, which recommends miniaturization and portability, a point of potential improvement in the future to make this method even more complete.

### Determination of sorbate and benzoate in mustard, ketchup and tomato sauce

The validated method was applied for the analysis of sorbate and benzoate in 30 commercial samples of mustard, ketchup and tomato sauce from different manufacturers. The results are shown in Table 4.

**Table 4:** Mass fractions of potassium sorbate and sodium benzoate in mustard, ketchup and tomato sauce samples

Sample	Preservative declared on the label	*w*(sorbate)/(mg/kg)	*w*(benzoate)/(mg/kg)
Mustard A	-	<LOD	<LOD
Mustard B	sodium benzoate	<LOD	180±15
Mustard C	sodium benzoate	<LOD	946±79
Mustard D	sodium benzoate	<LOD	559±85
Mustard E	sodium benzoate	<LOD	813±93
Mustard F	sodium benzoate	<LOD	621±48
Mustard G	potassium sorbate	607±10	917±14
Mustard H	sodium benzoate	<LOD	952±45
Mustard I	sodium benzoate	<LOD	992±0.8
Mustard J	sodium benzoate	<LOD	404±6
Ketchup A	potassium sorbate	<LOD	586±33
Ketchup B	potassium sorbate	430±7	<LOD
Ketchup C	-	<LOD	<LOD
Ketchup D	sodium benzoate	<LOD	163.5±1.2
Ketchup E	potassium sorbate	695±38	<LOD
Ketchup F	potassium sorbate	444±22	<LOD
Ketchup G	potassium sorbate	741±39	1054±70
Ketchup H	potassium sorbate	605±10	<LOD
Ketchup I	sodium benzoate	<LOD	1479±36
Ketchup J	-	<LOD	<LOD
Tomato sauce A	sodium benzoate	<LOD	362±79
Tomato sauce B	potassium sorbate	<LOD	352±34
Tomato sauce C	sodium benzoate	414±44	<LOD
Tomato sauce D	sodium benzoate	<LOD	326±18
Tomato sauce E	-	<LOD	<LOD
Tomato sauce F	potassium sorbate	589±66	<LOD
Tomato sauce G	potassium sorbate and sodium benzoate	431±19	447±21
Tomato sauce H	-	<LOD	<LOD
Tomato sauce I	-	<LOD	<LOD
Tomato sauce J	potassium sorbate	849±24	<LOD

LOD=limit of detection, LOD sorbate: *γ*=0.3 mg/L, LOD benzoate: *γ*=0.9 mg/L, -=label informed ’no preservatives’

Among the analyzed samples, six of them declared ’no preservatives’ on the label, and this claim was confirmed experimentally. Also, of the thirty evaluated samples, seven showed some kind of non-compliance. Significant problems involving mislabeling and non-compliance with the Brazilian legislation were found in the ketchup samples, where four out of ten assessed samples had some irregularity.

The Brazilian legislation only allows the addition of sorbic acid and its salts at the maximum mass fraction of 1000 mg/kg in ketchup (*10*). However, four analyzed samples contained sodium benzoate as a preservative. Ketchup samples A and G reported the use of sorbate on their label, although benzoate was detected experimentally. On the contrary, ketchup samples D and I declared benzoate on the ingredient list, despite the regulatory recommendation. Besides these mislabelings, ketchup samples G and I had benzoate mass fractions ((1054±70) and (1479±36) mg/kg, respectively) higher than the maximum limit of 1000 mg/kg established by the European Commission (*9*), which represents an infringement if these products are marketed in Europe. All ketchup samples that contained sorbate followed the regulatory limit of both legislations.

Regarding tomato sauce, all samples were in accordance with the limits established by the European and Brazilian legislation that allows the use of sorbate and benzoate at mass fractions of 1000 mg/kg (*9*, *10*). Experimentally obtained results confirmed that tomato sauce samples B and C also contained mislabeling related to the presence of sorbate and benzoate in the ingredient list.

For mustard, European and Brazilian legislation allows the addition of sorbate and benzoate (*9*, *10*), but in nine out of ten evaluated samples only benzoate was detected. The mass fractions of sorbate and benzoate in all samples were in accordance with the legislation (1000 mg/kg) and with their label.

Despite the compliance of almost all investigated samples with the European and Brazilian legislations, the sorbate and benzoate mass fractions found were, in general, higher than those reported in other studies. In Austrian mustards, the mean mass fraction of sorbic acid was 118.9 mg/kg and of benzoic acid 195.5 mg/kg, while in ketchup and tomato puree, the mean mass fraction of sorbic and benzoic acids were 486.0 and 788.7 mg/kg, respectively (*32*). Low mass fractions of sorbic and benzoic acids were also reported in Chinese ketchup at 175.9 and 307.6 mg/kg, respectively (*13*). In Iranian ketchup, sorbate and benzoate are not allowed, but Javanmardi *et al.* (*12*) found benzoate mass fractions of up to 38.7 mg/kg, however, none of them were detected in tomato paste (*2*). In contrast, 23.8% samples of Turkish ketchup showed a sum of sorbic and benzoic acids >1000 mg/kg (*33*).

Our findings reinforce the importance of the constant monitoring of sorbate and benzoate mass fractions in these products, especially ketchup, associated with the labeling inspection since children and young people are frequent consumers of these products, and their excessive intake can represent health risks, especially for these consumers (*7*). In addition, the fact that the sorbate and benzoate mass fractions found in this study were, in general, higher than those reported in other works lights up a warning signal about the possible excessive intake of these preservatives by Brazilian consumers since they are present in many different food products. Therefore, the constant monitoring and the use of allowed sorbate and benzoate mass fractions in mustard, ketchup and tomato sauce are indispensable to ensure the lower risk to the health of their consumers.

## CONCLUSIONS

In this study, the proposed methodology employing a green sub-minute capillary zone electrophoresis was validated and showed to be suitable for monitoring sorbate and benzoate mass fractions in mustard, ketchup and tomato sauce, with the advantages of using a simple, fast and solvent-free sample preparation procedure. Our findings indicated that of the 30 evaluated samples, six showed some kind of non-compliance with European or Brazilian legislation by containing a prohibited preservative or being mislabeled, where two of these samples (ketchup) contained benzoate, an analyte that is not allowed in this product by Brazilian legislation, at a mass fraction higher than 1000 mg/kg. Therefore, these aspects highlight the need for this kind of assessment, especially by using fast and reliable methods.
